# Regulation of the Omega-3 Fatty Acid Biosynthetic Pathway in Atlantic Salmon Hepatocytes

**DOI:** 10.1371/journal.pone.0168230

**Published:** 2016-12-14

**Authors:** Marte Avranden Kjær, Bente Ruyter, Gerd Marit Berge, Yajing Sun, Tone-Kari Knutsdatter Østbye

**Affiliations:** 1 Nofima, Ås, Norway; 2 Nofima, Sunndalsøra, Norway; Universidade de Vigo, SPAIN

## Abstract

Limited availability of the n-3 fatty acids EPA and DHA have led to an interest in better understanding of the n-3 biosynthetic pathway and its regulation. The biosynthesis of alpha-linolenic acid to EPA and DHA involves several complex reaction steps including desaturation-, elongation- and peroxisomal beta-oxidation enzymes. The aims of the present experiments were to gain more knowledge on how this biosynthesis is regulated over time by different doses and fatty acid combinations. Hepatocytes isolated from salmon were incubated with various levels and combinations of oleic acid, EPA and DHA. Oleic acid led to a higher expression of the Δ6 fatty acid desaturase (fad) genes Δ*6fad_a*, Δ*6fad_b*, Δ*6fad_c* and the elongase genes *elovl2* compared with cells cultured in medium enriched with DHA. Further, the study showed rhythmic variations in expression over time. Levels were reached where a further increase in specific fatty acids given to the cells not stimulated the conversion further. The gene expression of Δ*6fad_a_*and Δ*6fad_b* responded similar to fatty acid treatment, suggesting a co-regulation of these genes, whereas Δ*5fad* and Δ*6fad_c* showed a different regulation pattern. EPA and DHA induced different gene expression patterns, especially of Δ*6fad_a*. Addition of radiolabelled alpha-linolenic acid to the hepatocytes confirmed a higher degree of elongation and desaturation in cells treated with oleic acid compared to cells treated with DHA. This study suggests a complex regulation of the conversion process of n-3 fatty acids. Several factors, such as that the various gene copies are differently regulated, the gene expression show rhythmic variations and gene expression only affected to a certain level, determines when you get the maximum conversion of the beneficial n-3 fatty acids.

## Introduction

The polyunsaturated fatty acid (PUFA) bioconversion pathway in salmon is similar with that of the majority of other vertebrates, involving an alteration of desaturation and elongation steps. However, the pathway is still not completely elucidated in salmon. One Δ5 fatty acid (FA) desaturase (fad) gene and three Δ6fad genes (Δ*6fad_a*, Δ*6fad_b*, and Δ*6fad_c*) are cloned from salmon [1–3]. The salmon Δ5fad enzyme was primarily characterized as an n-3 Δ5 desaturase with low level of Δ6 and n-6 Δ5 activities [2]. The first Δ6fad gene characterized (later named Δ*6fad_a*) possesses predominantly Δ6 desaturase activity [1]. Monroig et al. [3] showed through functional characterization by heterologous expression in yeast that the cDNAs for the Δ*6fad_b* and Δ*6fad_c* genes only had Δ6 activity. In some marine fish species, the Δ6 enzyme has been found to also have Δ8 activity [4]. Further, Monroig et al. found that both Δ*6fad_a* and Δ*6fad_b* genes were highly expressed in intestine (pyloric caeca), liver and brain, whereas Δ*6fad_c* transcript was found predominantly in brain, with lower expression levels in all other tissues [3]. Modulations of desaturase gene expression and enzyme activities by both nutritional factors and environmental factors have been reported [3,5]. Three elongase genes have been cloned and characterised in salmon. The *SalElo* gene, now termed *elovl5a*, showed broad substrate specificity for PUFAs with a range of chain lengths, with the rank order being C_18_ > C_20_ > C_22_ [2]. More recently, reports on the cloning of two new elongase cDNAs: a second *elovl5b* elongase and an *elovl2-*like elongase have been published. Heterologous expression in yeast showed that the salmon Elovl5b elongated C18 and C20 PUFA, with low activity towards C22, while Elovl2 elongated C20 and C22 PUFA with lower activity towards C18 PUFA [6]. The elongase genes also responded to changes in dietary FA composition [6]. The final step in the bioconversion pathway includes chain shortening by peroxisomal β-oxidation [7]. Recently it was shown that some species of marine fish such as rabbitfish (*Siganus canaliculatus*) and Senegalese sole (*Solea senegalensis*) have Δ4 desaturases that would enable a more direct route for the synthesis of DHA [8,9], but this is not seen in salmon. Increased acyl-CoA oxidase (ACO) enzyme activity and *ACO* gene expression has been shown as a result of high docosahexaenoic acid (DHA, 22:6n-3) levels [10]. Stimulated peroxisomal β-oxidation by DHA has also been confirmed in other studies with Atlantic salmon [11]. The link between this step and the PUFA bioconversion pathway and long chain (LC) n-3 production is very scarcely studied.

It is hypothesised that salmonid aquaculture may be capable of becoming a net producer of LC n-3 FAs [12]. Atlantic salmon are capable of converting α-linolenic acid (ALA, 18:3n-3) to eicosapentaenoic acid (EPA, 20:5n-3) and DHA, but the conversion is not very efficient [13–16]. Therefore, their essential FA requirements are not met by C18 FAs alone, and must be provided with some dietary EPA and DHA in order to obtain good growth and health [13,14]. The optimal ratio between fish oil (FO) and vegetable oil in the diet, however, remain unknown.

The n-3 PUFA production is regulated, at least in part, by the substrate level of 18:2n-6 and 18:3n-3 [17]. In addition, oleic acid (OA, 18:1n-9) can stimulate the Δ5- and Δ6fads. This FA is the most abundant FA in rapeseed oil, which is the most common oil substitute in today’s aquaculture. The capacity for conversion of ALA to EPA and DHA, and the gene expression of the elongases, the Δ5- and Δ6fad activities in salmon is increased when fed high dietary levels of vegetable oils, while FOs depressed the capacities [6,18–20]. Similar results have been found by Zheng et al. [21], showing that EPA suppressed LC-PUFA synthesis in salmon cells and also suppressed the activity of the salmon Δ6fad promoter. High dietary levels of ALA might inhibit its own conversion to DHA in Atlantic salmon [13,14]. These results underpin the fact that the dietary level must be optimised to find the ideal feed for healthy and nutritious salmon. Finding the optimal ratio of FAs, stimulating the desaturation and elongation processes the most, would enable a more efficient use of vegetable oils (FAs) in the feed.

The overarching hypothesis of the present experiments is therefore that understanding the molecular basis of LC-PUFA biosynthesis and regulation will allow optimisation of the pathway.

## Materials and Methods

### Ethical statement

Rearing and slaughtering were conducted at Norwegian University of Life Sciences (Norway), which is approved by Norwegian Animal Research Authority (NARA). Stunning and sampling of fish were performed in accordance with the Norwegian Animal Welfare act. Tissue sampling was done only after fish were put to death, hence, no NARA approval was required according to Dr. G Bæverfjord (Nofima), appointed by NARA.

### General description

**Fig 1:**
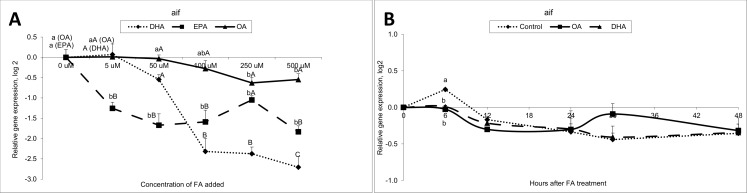
Gene expression results of apoptosis inducing factor gen from the time-course and dose response study. Data are presented as mean ± SEM (n = 3). Different letters indicate statistical differences (P≤0.05) between fatty acid treatments, within each time point, and between concentrations, within each fatty acid.

Atlantic salmon hepatocytes were used to study different aspects of the n-3 biosynthetic pathway. Three experiments were performed; (1) a time-course study over a 48h time period on the effect of FAs on gene expression, (2) a dose-response study on the effect of FAs on gene expression and (3) a ratio/metabolism study on the effect of different OA/n-3 FA ratios on FA profile, FA β-oxidation, elongation, desaturation and gene expression. In all three experiments, hepatocytes were incubated with non-radiolabelled OA, EPA and or DHA. In the ratio/metabolism study, hepatocytes were further incubated with radiolabelled ALA. The details of the different methodological and analytical steps are described below. The viability of the cells was evaluated through microscopy investigations and gene expression analysis of an apoptosis marker (Fig 1).

### Isolation of hepatocytes

Atlantic salmon of approximately 500 g (300 g– 950 g) (Norwegian University of Life Sciences, Norway) were kept in seawater at 8–10°C and fed on a commercial diet prior to isolation of hepatocytes. The fish were anaesthetized in Metacain (MS-222, Norsk Medisinaldepot, Norway). The abdominal cavity was exposed and the vena porta cannulated. The liver was perfused following a two-step collagenase procedure developed by Seglen [22] and modified by Dannevig and Berg [23], designed to obtain isolated hepatocytes. The liver was treated with collagenase (Type 1, Worthington 4197) and hepatocytes subsequently isolated by gentle shaking of the digested liver in Leibovitz`s L-15 medium (Gibco, Life Technologies, Paisley, UK). The suspension of parenchymal cells obtained in this manner was filtered through a 100 μm nylon filter. Hepatocytes were washed three times in Leibovitz`s L-15 medium and sedimented by centrifugation for 2 min at 107*g*. The hepatocytes were resuspended in growth media containing Leibovitz`s L-15 media with FBS (10%, PAA Laboratories, Australia), sodium bicarbonate (4.5 mM, Sigma-Aldrich, MO, USA), L-glutamine (2 mM, Sigma-Aldrich, MO, USA), Penicillin-Streptomycin solution (1%, PAA Laboratories, Australia) and Hepes (10 mM, Sigma-Aldrich, MO, USA). Cell viability was assessed by staining with Trypan Blue (0.4%, Sigma-Aldrich, MO, USA). Approximately 5 x 10^5^ hepatocytes / cm^2^ were plated onto cell culture wells coated with laminin (1.2 μl/cm^2^, Merck, Darmstadt, Germany), and left to attach overnight at 13°C. Details for each experiment are described below.

### Incubation of hepatocytes with fatty acids

The cells were incubated with OA, EPA and DHA (Sigma-Aldrich, MO, USA) alone or in different ratios. The FAs were added to the growth media (containing 2% FBS) in the form of their sodium salts bound to BSA (2.7/1, molar ratio). Briefly, 25 mg FA was dissolved in 1 ml chloroform (to 76 mM) and evaporated under a stream of nitrogen at 50–60°C or 37–40°C for OA and DHA/EPA, respectively. Preheated 0.1 M NaOH (3 ml) of was slowly added. The FA-NaOH solution was then transferred to 12 ml PBS-albumin, which contained 1.88 g albumin. The pH was adjusted to 7. Each solution was made as a stock solution of 5mM.

#### Time-course study

The cells in culture were supplemented with either OA or DHA or albumin (control) to a final concentration of 100 μM for the FAs. In total, cells were plated in forty-eight 9.6 cm^2^ wells, 15 parallels per FA treatment. Three wells with cells were harvested at time zero, and per treatment, three parallels were harvested 6 h, 12 h, 24 h, 30 h or 48 h after supplementation of FAs for gene expression studies.

#### Dose-response study

For the dose-response study, the cells were incubated with OA, EPA and DHA in five different concentrations (5 μM, 50 μM, 100 μM, 250 μM and 500 μM), each FA given to three parallel wells (9.6 cm^2^) per concentration. Cells in three wells were given albumin (control; 0.0025 g/ml). The cells were incubated for 24 h, and harvested for gene expression studies.

#### Ratio / metabolism study

In the ratio/metabolism study, the cells were added media with FAs of four different combinations (100 μM OA (100OA), 25 μM OA:75 μM DHA (25OA75DHA), 75 μM OA:25 μM DHA (75OA25DHA) or 100 μM DHA (100DHA)), each combination in 20 parallels. Control cells (15 wells) were added albumin (0.0025 g/ml). In total, cells were plated onto 95 wells (9.6 cm^2^). The cells were incubated for 66 h. After the incubation period, five parallel wells per FA treatment were harvested for gene expression analysis, further five parallels for ACO analysis and five parallels for FA analysis. Cells for metabolism analyses (β-oxidation, elongation and desaturation) were washed twice in PBS with 1% albumin, followed by one time in PBS. Further, the cells were incubated with 7 μM (final concentration) [1-14C]-18:3n-3 (American Radiolabel Chemicals Inc., MO, USA) for 24 h. The FA was added to the media in the form of its sodium salt bound to BSA.

### Lipid extraction and analysis of fatty acids

The total non-radiolabelled FA profiles in the hepatocytes from the ratio/metabolism study were determined from five parallels for each FA treatment. Total lipids were extracted from cells and their culture media by the method described by Folch et al. [24]. The chloroform phase was dried under N_2_ and the residual lipid extract was re-dissolved in chloroform. Further, they were trans-methylated overnight with 2,2-dimethoxypropane, methanolic HCl and benzene at room temperature, as described by Mason and Waller [25], and by Hoshi et al. [26]. The methyl esters of FAs thus formed were separated in a gas chromatograph (Hewlett Packard 6890) with a split injector, SGE BPX70 capillary column (length 60 m, internal diameter 0.25 mm and thickness of the film 0.25 μm), flame ionisation detector and HP Chem Station software. The carrier gas was helium. The injector and detector temperatures were 300°C. The oven temperature was raised from 50°C to 170°C at a rate of 4°C/min, and then raised to 200°C at a rate of 0.5°C/min, and finally to 300°C at a rate of 10°C/ min. The relative quantity of each FA present was determined by measuring the area under the peak in the GC spectrum corresponding to that FA.

The radiolabelled FAs of the cells were determined by reversed phase high pressure liquid chromatography (HPLC) as described by Narce et al. [27]. The mobile phase was acetonitrile:water (85:15 v/v) at a flow rate of 1 ml/min and a temperature of 30°C. The column used was a symmetry 3.5 μm C_18_ column (Waters) and the FAs were detected with a radioactive flow-detector A-100 (Radiomatic Instrument & Chemicals, Tampa, FL, USA). The FA were identified by comparing the sample retention times with the retention times of FA standards (American Radiolabel Chemicals Inc., MO, USA). Some of the FA standards were nonradioactive (Sigma-Aldrich, MO, USA) and therefore their absorbance was measured in a UV detector (Waters 2996 PDA Detector) at 215 nm.

### Beta oxidation

The β-oxidation occurring in the cells was measured by determination of the ^14^C-containing oxidation products, acid soluble products (ASP) and CO_2_ as described by Christiansen et al. [28]. ^14^C-CO_2_ produced during the incubation of the cells was measured by transferring 1.4 ml of the cell media to glass vials sealed with a rubber stop and a central well containing a Whatman filter paper with 0.3 ml phenylethylamine/methanol (1:1 v/v). The medium were acidified with 0.3 ml 1 M HClO_4_ and ^14^C-CO_2_ was trapped for 1 h. Then the filter papers were placed in vials and dissolved in 8 ml of scintillation fluid (Ecoscint^TM^ A scintillation solution, National Diagnostics, Atlanta, USA) for scintillation counting (Radiomatic Instrument & Chemicals, Tampa, FL, USA). The amount of ^14^C-ASP was determined by adding 0.5 ml ice cold 2 M HCIO_4_ to 1 ml of the incubation medium and incubated at 4°C for 1 h. The samples were centrifuged at 17,950×*g* for 10 min at 4°C and 100 μl of the supernatant was collected for scintillation counting (Radiomatic Instrument & Chemicals, Tampa, FL, USA).

### Acyl CoA Oxidase assay

ACO (EC 1.3.3.6) activity was assayed in hepatocytes from the ratio/metabolism study by determining the rate at which hydrogen peroxide was produced, coupled to the oxidation of 2’,7’- dichlorofluorescine, essentially as described by Small et al. [29]. The oxidation of 2’,7’-dichlorofluorescine by hydrogen peroxide to 2’,7’-dichlorofluorescein was followed spectrophotometrically at 502 nm in a SPECTROstar Nano plate reader (BMG Labtech). The reaction mixture contained 0.1 M Tris-HCl (pH 8.5), 0.05 mM 2’,7’-dichlorofluorescine, 0.05 mg/ml horseradish peroxidase type II, 0.02 mM FAD, 0.6 mg/ml BSA and 0.002% Triton-X 100, and was started with 0.6 mM palmitoyl-CoA. All concentrations are given as final values. The ACO activity was calculated asnmol ACO * minute ^-1^ * mg protein^-1^.

### Protein measurements

Protein concentration was determined using a total protein kit (Micro Lowry/Peterson’s modification, Sigma-Aldrich, MO, USA) based the method of Lowry and modified by Peterson [30,31]. Standard specimens were prepared by making a series of dilutions of BSA in water. Sodium chloride was added to a final concentration of 0.1 M, in order to reduce ampholyte interference. The protein present was precipitated by adding 0.1% trichloracetic acid (TCA) in the presence of 0.15% deoxycholate (DOC). Colour was measured at 500 nm in a SPECTROstar Nano plate reader (BMG Labtech).

### RNA isolation and cDNA synthesis

Total RNA was isolated from hepatocytes using an RNeasy® Mini Kit (Qiagen, CA, USA) following the manufacturer`s protocol. Samples were first lysed in RNeasy Lysis Buffer (RLT) and then homogenized by using QIAshredder columns (Qiagen, CA, USA). Residual amounts of DNA were removed using an RNase-Free DNase Set (Qiagen, CA, USA) during the RNeasy procedure. The total RNA concentrations were determined using NanoDrop® ND-1000 Spectrophotometer (Thermo Scientific, DE, USA). Thereafter, AffinityScript QPCR cDNA Synthesis Kit (Agilent Technologies, CA, USA) was used to synthesis cDNA according to the manufacturer`s protocol, using 500 ng RNA in a total reaction volume of 10 μl (1x cDNA synthesis master mix, 15 ng/uL oligo(dt), 1 uL AffinityScript RT/ RNase Block enzyme mixture). The cDNA synthesis was performed with 5 min primer incubation at 25°C, 45 min RT step at 42°C, and 5 min of RT inactivation at 95°C.

### Sequence information and primer design

Vector NTI Advance 10 (Life Technologies, Paisley, UK) was used to design real-time PCR primers based on available salmon sequences in the Genbank^®^. The primers were purchased from Life Technologies (Paisley, UK).

### Real-time PCR

Real-time PCR was performed in a LightCycler 480 Instrument (Roche Applied Science, Germany) with gene-specific primers (Table 1). RNA polymerase 2 (*rpol2*), elongation factor 1 alpha (*ef1α*), *β-actin*, NADH-ubiquinone oxidoreductase (*nour*) and eukaryotic translation initiation factor 3 (*etif*) were evaluated as reference genes by Genorm [32]. *Rpol2* (time-course study), *β-actin* (dose-response study) and *etif* (ratio/metabolisme study) were found to be the most stable genes in the respective cell trials. Standard curves for each primer pair were run to calculate primer efficiencies (*E*). The PCR master mix consisted of 1 μl forward and reverse primer (final concentrations of 0.5 μM), 4 μl of a 1:10 dilution of cDNA and 5 μl LightCycler 480 SYBR Green I Master (Roche Applied Science, Germany). All samples were analysed in parallels with a non-template control for each gene. The reaction conditions were 95°C for 5 min, 45 cycles of 95°C for 15 seconds and 60°C for 1 min. A melting curve analysis (95°C for 5 seconds and 65°C for 1 min, 97°C) was run to confirm the presence of a single PCR product. The relative gene expression level was calculated according to the ΔΔCt method and adjusted for differences in primer efficiency [33].

**Table 1:** Primers for Quantitative Real-Time PCR Analysis.

Gene	Accession no.	Direction	Primer sequence 5´→ 3´
*rpol2*	CA049789	Forward	TAACGCCTGCCTCTTCACGTTGA
		Reverse	ATGAGGGACCTTGTAGCCAGCAA
*ef1α*	AF321836	Forward	CACCACCGGCCATCTGATCTACAA
		Reverse	TCAGCAGCCTCCTTCTGAACTTC
*β-actin*	AF012125	Forward	ACATCAAGGAGAAGCTGTGC
		Reverse	GACAACGGAACCTCTCGTTA
*nour*	DW532752	Forward	CAACATAGGGATTGGAGAGCTGTACG
		Reverse	TTCAGAGCCTCATCTTGCCTGCT
*etif*	DW542195	Forward	CAGGATGTTGTTGCTGGATGGG
		Reverse	ACCCAACTGGGCAGGTCAAGA
*Δ5fad*	AF478472	Forward	GCTTGAGCCCGATGGAGG
		Reverse	CAAGATGGAATGCGGAAAATG
*Δ6fad_a*	AY458652	Forward	TCCCCAGACGTTTGTGTCAGATGC
		Reverse	GCTTTGGATCCCCCATTAGTTCCTG
*Δ6fad_b*	GU207400	Forward	TGACCATGTGGAGAGTGAGGG
		Reverse	AACTTTTGTAGTACGTGATTCCAGCT
*Δ6fad_c*	GU207401	Forward	TGAAGAAAGGCATCATTGATGTTG
		Reverse	CACAAACGTCTAGGAAATGTCC
*elovl2*	TC91192	Forward	CGGGTACAAAATGTGCTGGT
		Reverse	TCTGTTTGCCGATAGCCATT
*elovl5a*	NM_001123567	Forward	ACAGTAACCCCAGAGACCCA
		Reverse	TTGTCCCCACCACACTGAAG
*elovl5b*	NM_001136552	Forward	GCAACCTTGACCCAAACAGG
		Reverse	CCTTGTCTCTACGCAAGGGA
*aif*	TC37490	Forward	AGGTGGAGTCCCAAGGAATCTGC
		Reverse	CCCCAAGAAACCTCCTCCAATG
*aco*	DQ364432	Forward	CCTTCATTGTACCTCTCCGCA
		Reverse	CATTTCAACCTCATCAAAGCCAA

*rpol2 =* RNA polymerase 2, *ef1α* = elongation factor 1 alpha, *nour* = NADH-ubiquinone oxidoreductase, *etif* = eukaryotic translation initiation factor 3, *Δ5fad = Δ*5 fatty acid desaturase, *Δ6fad_a = Δ*6 fatty acid desaturase a, *Δ6fad_b = Δ*6 fatty acid desaturase b, *Δ6fad_c = Δ*6 fatty acid desaturase *c*, *elovl2* = elongase 2, *elovl5a* = elongase 5a, *elovl5b* = elongase *5b*, *aif =* apoptosis inducing factor, *aco =* acyl-CoA oxidase

### Statistical analyses

All the data were subjected to a one-way analysis of variance (ANOVA). Significant effects were indicated at a 5% level. The differences were ranked using Duncan’s Multiple Range Test. Statistical analyses were conducted using the software package UNISTAT (London, England).

## Results

### Time-course study

**Fig 2:**
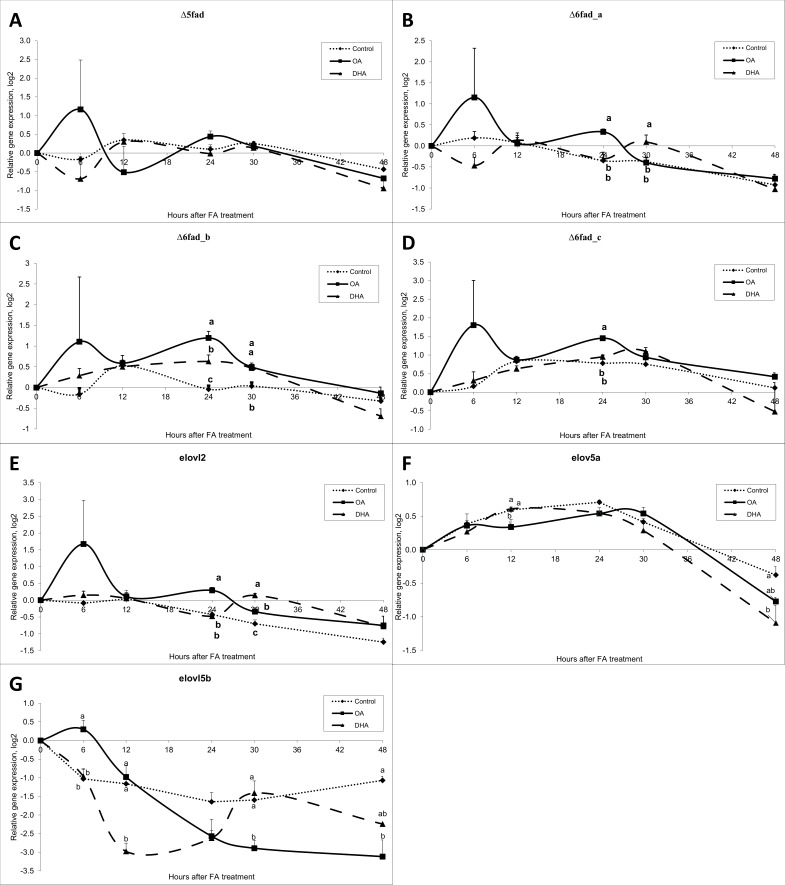
Gene expression results of desaturase- and elongase genes from the time-course study. Data are presented as mean ± SEM (n = 3). Different letters indicate statistical differences (P≤0.05) between fatty acid treatments, within each time point.

Differences in expression (during 48 h time course) of genes related to the elongation and desaturation processes were analysed at different time points after FA treatment (Fig 2, Table 2). Already after 6 h, we could see a response in all genes analysed, however, the expression showed huge variations, and none of the differences were significant at this time point. Interestingly, when the cells were added DHAs, the genes *Δ5fad* (Fig 2A) and *Δ6fad_a* (Fig 2B) showed a decreased expression 6 h after the treatment followed by an increased expression 12 h after treatment. The expression was decreased again 18 h after treatment, and once more followed by an increased expression 24 h after treatment. After 30 h, the expression seemed to decrease until the end of our study. A similar pattern was seen for the *elovl2* gene expression (Fig 2E). *Δ6fad_b* (Fig 2C) and *Δ6fad_c* (Fig 2D), increased their expression until 24 h after treatment, followed by a decrease as a response on DHA treatment. OA treatment gave the same variation pattern on the desaturase genes and *elovl2*, however, the response was opposite as for DHA treatment, increasing after 6 h, followed by a decrease, then increasing, and finally decreasing after 24 h. In addition, the control cells, not receiving any FAs, had variations in their gene expression over time, indicating a natural variation pattern. OA treatment resulted in significantly higher expression of the genes *Δ6fad_a*, *Δ6fad_b*, *Δ6fad_c* and *elovl2* (Fig 2B–2E), compared to control treatment 24 h after addition of FAs. The two elovl5 isomers, *elovl5a* and *elovl5b*, did not show similar gene expression pattern. *elovl5a* increased the expression from first time-point and showed stable expression until a down-regulation 30 h after initiation of treatment. *elovl5b*, on the other hand, decreased gene expression after treatment (except for OA at 6 h) and was kept low for all treatments.

**Table 2:** Summary of the Fatty Acid Response on Elongase and Desaturase Genes.

	*Δ5fad*	*Δ6fad_a*	*Δ6fad_b*	*Δ6fad_c*	*elovl2*	*elovl5a*	*elovl5b*
**OA**		↑	↑	↑	↑	↑↓	↓
**EPA**	↓		↓		↑	↑↓	↓
**DHA**	↓	↑↓	↑↓		↑	↑	↓

↓Down regulation; ↑Up regulation

### Dose-response study

**Fig 3:**
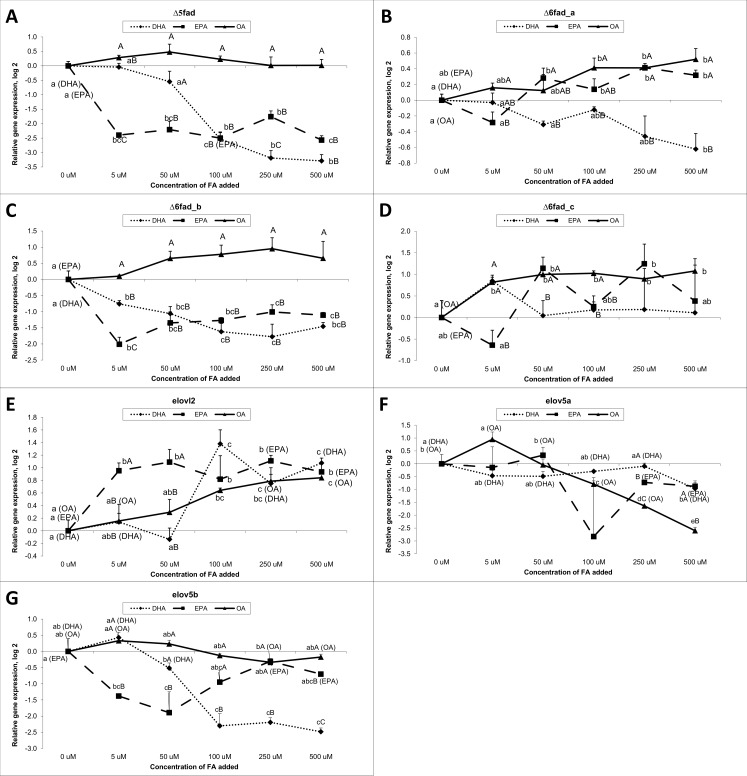
Gene expression results of desaturase- and elongase genes from the dose-response study. Data are presented as mean ± SEM (n = 3). Different letters indicate statistical differences (P≤0.05) between concentrations, within each fatty acid.

Treatment of hepatocytes with increasing concentrations of FAs (0 μM, 5 μM, 50 μM, 100 μM, 250 μM and 500 μM) for 24 h showed that cells responded by increasing or decreasing the expression of genes related to the elongation and desaturation pathway (Fig 3, Table 2). The *Δ5fad* gene expression (Fig 3A) tended to increase with OA treatment, while EPA and DHA treatment, on the other hand, down-regulated *Δ5fad* gene expression relative to control. With EPA treatment, the expression of *Δ5fad* levelled off after 5 μM FA added, and with DHA after 100 μM FA added. The exact same expression pattern was observed for the *Δ6fad_b* gene (Fig 3C). The *Δ6fad_a* gene (Fig 3B) responded on OA treatment by increasing gene expression and on DHA treatment by decreasing expression relative to control as with the two former genes mentioned. The gene expression of *Δ6fad_c* was increased by OA treatment relative to control after 5 μM FA added, however the expression did not continue to increase with increasing OA concentration given to the cells. The *Δ6fad_c* expression was not affected by EPA or DHA treatment in any clear direction. The elongase2 gene was up-regulated by all three FA treatments (Fig 3E). The expression of *elovl2* increased with increasing OA concentrations up to 500 μM. The expression increased up to 100 μM added DHA and 5 μM added EPA. Interestingly, the dose-response study demonstrated that the increasing concentration of FAs added to the cells was affecting the expression of the genes only until a certain point. Gene expression of *elov5a* was up-regulated by 5 μM OA added, but higher concentration of OA seemed to down-regulate the expression relative to the control (Fig 3F). EPA and DHA (except for 500 uM) did not significantly affect the *elov5a* gene expression compared to the control. DHA (50–500 μM) and low concentrations of EPA (5 and 50 μM) down-regulated *elov5b* expression relative to control, whereas different concentrations of OA induced minor changes on *elov5b* gene expression (Fig 3G).

### Ratio / metabolism study

Hepatocytes were pre-treated with FAs of four different combinations of OA and DHA to change their endogenous FA composition and to further study how this changed composition would affect the conversion pathway and the actual conversion of ALA. Addition of FAs to the cells changed their FA profile, however, not very profoundly. The FA compositions in hepatocytes reflected the ratio of OA and DHA given to the hepatocytes (Table 3). Incubation with increasing level of OA led to increasing percentage of 18:1n-9 in hepatocytes. The percentage of n-3 FAs, on the other hand, was increased with DHA incubation. The percentage of EPA and DHA were increased from 17.5% in 100OA incubation to 20% in 75OA25DHA incubation and to approximately 22% in 25OA75DHA and 100DHA incubation.

**Table 3:** Fatty Acid Composition in Hepatocytes.

Fatty acids (% of total)	100OA					75OA25DHA					25OA75DHA					100DHA					Control			
**Saturated FA**																								
**C 14:0**	1.1	±	0.12			1.0	±	0.04			1.1	±	0.01			1.1	±	0.02			1.1	±	0.05	
**C 16:0**	10.2	±	0.37	^a^		10.3	±	0.08	^ab^		10.9	±	0.05	^c^		11.2	±	0.13	^c^		10.8	±	0.05	^bc^
**C 17:0**	0.5	±	0.10			0.6	±	0.04			0.5	±	0.12			0.6	±	0.07			0.3	±	0.25	
**C 18:0**	5.2	±	0.08	^a^		5.1	±	0.04	^a^		5.5	±	0.04	^bc^		5.5	±	0.03	^b^		5.7	±	0.09	^c^
**Monoenes**																								
**C 16:1 n-7**	3.0	±	0.05			3.0	±	0.04			2.8	±	0.06			2.9	±	0.06			2.6	±	0.33	
**C 18:1 n-7**	2.9	±	0.04	^ab^		2.9	±	0.02	^a^		2.9	±	0.01	^a^		3.0	±	0.03	^b^		3.2	±	0.01	^c^
**C 18:1 n-9**	35.1	±	0.24	^a^		34.1	±	0.11	^b^		30.9	±	0.06	^c^		30.3	±	0.26	^d^		32.0	±	0.07	^e^
**C 20:1 n-9**	5.0	±	0.07	^d^		4.7	±	0.02	^c^		4.1	±	0.02	^a^		4.2	±	0.09	^a^		4.5	±	0.04	^b^
**C 22:1 n-7**	0.5	±	0.07			0.5	±	0.02			0.6	±	0.02			0.6	±	0.02			0.5	±	0.02	
**n-6 FAs**																								
**C 18:2 n-6**	6.7	±	0.06	^a^		6.7	±	0.03	^a^		6.8	±	0.02	^a^		6.7	±	0.10	^a^		7.2	±	0.07	^b^
**C 20:2 n-6**	1.7	±	0.02	^a^		1.8	±	0.06	^b^		1.8	±	0.01	^b^		1.9	±	0.02	^bc^		1.9	±	0.03	^c^
**C 20:3 n-6**	1.0	±	0.02	^ab^		1.0	±	0.01	^b^		0.9	±	0.02	^a^		1.0	±	0.03	^ab^		1.2	±	0.07	^c^
**C 20:4 n-6**	2.1	±	0.05	^a^		2.2	±	0.03	^ab^		2.3	±	0.01	^b^		2.2	±	0.06	^ab^		2.5	±	0.03	^c^
**Σ N-6**	**11.9**	±	**0.11**	^**a**^	** **	**12.0**	**±**	**0.07**	^**ab**^	** **	**12.1**	**±**	**0.01**	^**b**^	** **	**12.0**	**±**	**0.08**	^**ab**^	** **	**13.1**	**±**	**0.04**	^**c**^
**n-3 FAs**																								
**C 18:3 n-3**	1.6	±	0.04	^abc^		1.6	±	0.00	^a^		1.7	±	0.02	^c^		1.6	±	0.04	^ab^		1.7	±	0.03	^bc^
**C 20:3 n-3**	0.4	±	0.01	^a^		0.5	±	0.01	^b^		0.5	±	0.01	^c^		0.5	±	0.01	^c^		0.6	±	0.02	^c^
**C 20:5 n-3**	1.6	±	0.01	^a^		1.7	±	0.01	^b^		2.0	±	0.02	^c^		2.0	±	0.02	^c^		1.6	±	0.03	^a^
**C 22:5 n-3**	0.8	±	0.02	^a^		0.8	±	0.02	^a^		1.0	±	0.03	^c^		1.0	±	0.03	^bc^		0.9	±	0.01	^b^
**C 22:6 n-3**	16.0	±	0.28	^a^		18.1	±	0.09	^b^		20.0	±	0.07	^c^		20.2	±	0.26	^c^		17.6	±	0.22	^b^
**Σ N-3**	**20.9**	±	**0.38**	^**a**^		**23.0**	**±**	**0.10**	^**b**^		**25.5**	**±**	**0.08**	^**c**^		**25.6**	**±**	**0.25**	^**c**^		**22.7**	**±**	**0.23**	^**b**^
**Σ EPA/DHA**	**17.5**	±	**0.28**	^**a**^		**19.8**	**±**	**0.10**	^**b**^		**21.9**	**±**	**0.06**	^**c**^		**22.2**	**±**	**0.28**	^**c**^		**19.2**	**±**	**0.22**	^**b**^

The quantity of each fatty acid is given in percent of total fatty acids. Data are means (n = 5) ± SEM. Different letters denote significant differences between the dietary groups, within each fatty acid. Fatty acids constituting less than 0.5% of total fatty acids are not included in the table.

**Fig 4:**
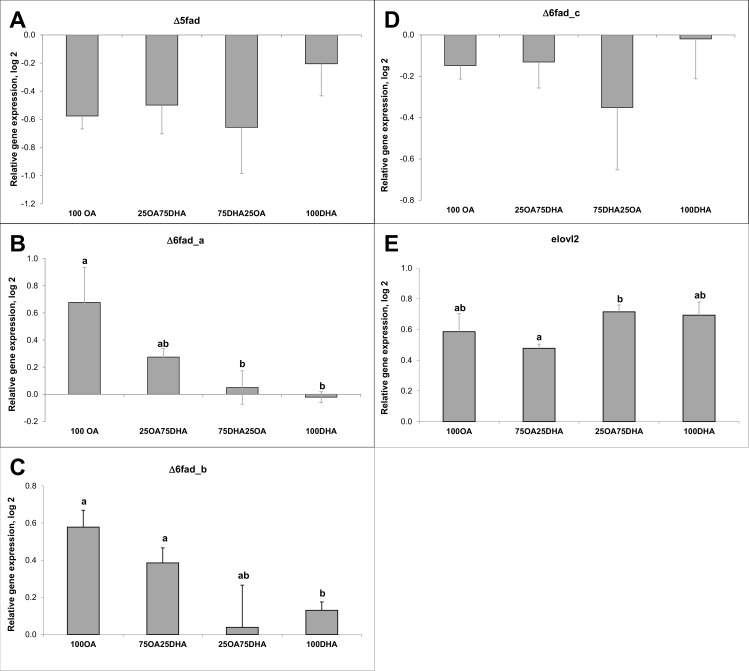
Gene expression results of desaturase- and elongase genes from the ratio/metabolism study. Data are presented as mean ± SEM (n = 5). Different letters indicate statistical differences (P≤0.05).

Altered endogen FA ratio did not affect the *Δ5fad* and *Δ6fad_c* gene expression (Fig 4). The *Δ6fad_a* and *Δ6fad_b* gene expression, on the other side, showed that the expression decreased with increasing amount of DHA/decreasing amount of OA in the cells. As in the dose-response study, the *elovl2* gene was up-regulated by high DHA levels.

**Fig 5:**
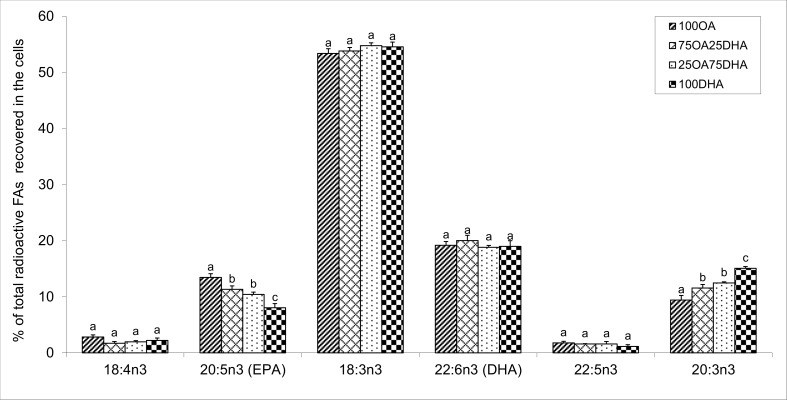
Metabolism of [1-^14^C]18:3 n-3. Data are presented as mean ± SEM (n = 5). Different letters indicate statistical differences (P≤0.05) between treatment groups, within each fatty acid.

Addition of radiolabelled ALA to the hepatocytes revealed an actual elongation and desaturation (Fig 5). However, the major quantity of radiolabelled n-3 FAs found in hepatocytes was predominantly unmetabolised [1-^14^C]-18:3n-3 substrate (more than 50%). About 45% of added [1-^14^C]-18:3n-3 was metabolised, 20% was converted all the way to DHA. However, only minor differences were found between the different treatment groups. The amount of EPA formed was increasing significantly with increasing level of OA / decreasing level of DHA in the cells. The intermediate product, 20:3n-3 (elongation product of 18:3n-3) increased with increasing level of f DHA in the cells.

**Fig 6:**
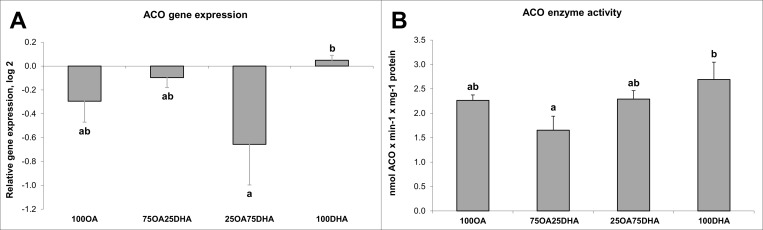
**(A) ACO gene expression. (B) ACO enzyme activity.** Data are presented as mean ± SEM (n = 5). Different letters indicate statistical differences (P≤0.05).

Minor amounts of added [1-^14^C]-18:3n-3 was oxidised, only approximately 0.2% of added lipids (Table 4). ACO seemed to be up-regulated by high DHA levels (Fig 6), the *ACO* gene expression was highest in the cells given 100% DHA. Correspondingly, the ACO enzyme showed the highest activity in the cells given 100% DHA.

**Table 4:** Distribution of Radioactivity in the Cells and the Medium after Incubation of Hepatocytes with [1-^14^C]18:3 n-3 for 24 H.

	100 OA				75OA25DHA				25OA75DHA				100DHA			
**CO2 in medium** (nmol mg protein ^-1^)	0,020	±	0,001		0,024	±	0,003		0,018	±	0,001		0,022	±	0,003	
**Cellular lipids** (nmol mg protein ^-1^)	1,30	±	0,04		1,27	±	0,16		1,03	±	0,08		1,34	±	0,22	
**Total uptake** (nmol mg protein ^-1^)	1,32	±	0,04		1,29	±	0,16		1,05	±	0,08		1,36	±	0,22	
**Oxidised lipid** (% of lipids taken up)	1,51	±	0,06	^a^	1,82	±	0,05	^c^	1,72	±	0,06	^bc^	1,63	±	0,05	^ab^

Data are presented as mean ± SEM (n = 5). Different letters indicate statistical differences (P≤0.05).

## Discussion

The time-course study showed that the genes in the LC-PUFA pathway had a rhythmic variation in expression pattern during the 48 h experiment. Effect of natural variations in the expression of genes in salmon has been scarcely studied. One recent study by Betancor et al. [34] showed however, that specific genes of lipid metabolism and homeostasis in liver of Atlantic salmon were under daily rhythm regulation. In mammals, FA desaturation has been shown to follow a circadian rhythm pattern [35,36]. Francis et al. [37] present results pointing towards the existence of cyclic mechanisms relative to FA utilization/retention in fish. The fish stored FAs with varying degree of efficiency depending on the time of the day when they were fed, and the Δ6fad activity was enhanced in fish subjected to weekly feeding alternation schedules. These results are interesting in the sense that it might be optimal to administrate a diet lower in LC n-3 FAs when EPA and DHA when the desaturation capacity is at its highest, and rather feed with vegetable sources as ALA. However, it might not be feasible for the aquaculture industry. On the other hand, the variations in expressions over time may explain discrepancies in the literature, and point to the importance of taking care of when to sample for RNA and what samples that are comparable. This might also explain the different result for some of the genes in the different cell trials performed in the present study. From the time-course study, the most significant differences were between treatments 24 h after FA addition (except for the *elovl5* isomers). OA increased the gene expression of the *∆*6fad isoforms and *elovl2* relative to both control and DHA in the hepatocytes. This time-point was further used in the dose-response study. The result from the time-course study show that other time-points may give different results.

The dose-response study revealed that OA increased the expression of the three *∆*6fad genes and the *elovl2* gene. DHA decreased the expression of *∆5fad*, *∆6fad_a*, *∆6fad_b* and *elovl5b*, while EPA decreased the *∆5fad* and *∆6fad_b* gene expression. Both EPA and DHA increased the expression of the *elovl2* gene. The dose-response study also demonstrated that an increasing concentration of FAs added to the cells affected the expression of the genes only until a certain point. Increasing the concentrations above certain levels did not seem to further up-regulate or down-regulate the genes. In similar manner, studies have shown that increasing dietary ALA elevates DHA, but only up to a maximum of 1% energy ALA [38]. These results suggest that the level of oils or FAs in the feed is an important factor for the outcome. We might reach levels where a further increase in specific FAs would not stimulate, but rather inhibit the conversion. This will be further discussed below.

Altered endogenous FA ratio in the cells also affected the *Δ6fad_a* and *Δ6fad_b* gene expression. The expression decreased with increasing amount of DHA/decreasing amount of OA. The *Δ5fad* and *Δ6fad_c* gene expression, on the other side, showed no change. There seems to be differences in the regulation between the different *Δ*6fad genes. The *Δ6fad_a* and *Δ6fad_b* gene expressions were increased by OA in the time-course study and the dose-response study, while in the dose-response study, EPA and DHA decreased their expression. The OA/DHA ratio also affected their expression in the similar manner. The *Δ6fad_c* gene expression was, however, not significantly affected. This is supported by findings of Monroig et al. [3] showing that the expression levels of the *Δ6fad_a* gene in liver and the *Δ6fad_b* gene in intestine were significantly higher in fish fed diets containing vegetable oil compared to fish fed FO, suggesting up-regulation in response to reduced dietary EPA and DHA. In contrast, no significant differences were found between transcript levels of *Δ6fad_c* in liver or intestine of fish fed vegetable oil compared to fish fed FO. Based on these observations it is plausible to hypothesize that *Δ6fad_b* may be regulated similarly to *Δ6fad_a*, and that *Δ6fad_c* is regulated differently. This prediction is also supported by the gene expression data of Monroig et al. [3], which showed substantial differences between *Δ6fad_c* and the other *Δ*6fad genes, with *Δ6fad_c* expression being very largely confined to brain whereas *Δ6fad_a* and *Δ6fad_b* was expressed in intestine, liver and brain. *Δ5fad* gene expression was not significantly affected by OA in any of our cell trials. Only in the dose-response study, EPA and DHA decreased the expression of the *Δ5fad* gene. This is supported by results found in another study from our group where different FAs had little effect on the gene expression of *Δ5fad* in muscle cells (Østbye et al., manuscript in prep). The different enzymatic activities of the *Δ*6fads and *Δ*5fad may all have distinct biological/physiological roles in salmon. Our results point in the same direction as proposed by Monroig et al. [3], that the same genes might be differentially regulated in different tissues. How this is regulated is an unknown question, but might be related to the expression, activities and regulation of transcription factors.

Hepatocytes incubated with increasing levels of OA showed an actual increased percentage of this FA and its elongation product 20:1n-9 in cellular lipids. In the hepatocytes incubated with increasing levels of DHA/decreasing levels of OA, the percentage of DHA and the sum of total n-3 FAs significantly increased. These results agree with findings from previous *in vivo* and *in vitro* studies in salmon, which show that tissue or hepatocyte FA composition is greatly influenced by the FAs supplemented to the diet or to the cell culture media [13,16,18,39–43]. Metabolism results showed that in the cells incubated with [1-14C]-18:3n-3, elongation and desaturation occurred. The main radiolabelled products were the desaturation product 18:4n-3 and the desaturation and elongation products EPA, docosapentaenoic acid (DPA, 22:5n-3) and DHA. The gene expression results, showing increased activity of elongation and desaturation genes with addition of OA, were supported by metabolism results. Cells with increased endogenous levels OA/decreased endogenous levels of DHA had significantly more EPA produced. In addition, recovery of un-metabolised [1-14C]-18:3n-3 substrate in the cells showed a weak tendency to decrease with increased percentage of OA in the cells, indicating that more of the [1-14C]-18:3n-3 substrate had been converted to other metabolic products in the groups with high endogenous level of OA than in those with high endogenous levels of DHA. There was also a tendency to higher production of 18:4n-3 in the high OA groups than in the high DHA groups. These results are in agreement with previous studies showing that vegetable oil can stimulate elongase and desaturase activity and/or gene expression [10,16,20,44,45]. No differences were found in DHA production between the four groups. One possible explanation could be that all groups had relatively high endogenous DHA levels, and high DHA levels are shown to inhibit its own production [46]. It is also noteworthy to mention that in another cell trial where actual elongation and desaturation were studied, FA composition of cells was not static [47]. Several days after addition of ALA, a reduction of EPA and an increase in DPA and DHA levels were apparent, clearly suggesting that a longer time period would have been required for allowing the complete bioconversion of ALA up to DHA [47]. In agreement with this observation, studies tracing ingested labelled-ALA found that DHA take longer time to accumulate in the plasma compared with EPA and DPA [48,49]. This may be another reason for low conversion to DHA in our study.

The *Δ*6fad step is generally considered to be the main rate-limiting step in the PUFA biosynthetic pathway. Recent studies have, however, suggested that rather than the existence of a single rate-limiting step affecting the overall pathway, a combination of different level of efficiency in each step is responsible for the production of n-3 LC-PUFA biosynthesis [47,50,51]. The efficiency of ALA bioconversion to EPA and DHA in hepatocytes has been commonly attributed to enzyme affinity, substrate availability and transcriptional factors in experiments assessing FA metabolism in the cells alone [17,52], but the presence of bioconversion products is also known to have direct effects. Tocher et al. [53] speculated that the rate of desaturation is a direct result of product reduction rather than an increased supply of precursors. It has also been suggested that high levels of LC n-3 PUFAs have a feedback inhibition effects on the enzymes of FA desaturation [45,46,54]. Other studies demonstrated that the rate of C18 FA desaturation was more strongly regulated by the competition between C18 substrates or the intermediate product 24:5n-3 for *Δ*6fad than by DHA [13,50,55–58]. Gibson et al. [56] conclude in a recent study that both the substrate FAs and the LC-PUFAs contribute to the regulation and that the ratio of ALA and DHA is very important for the outcome of desaturation and elongation. Others [57,59] have shown that an excess of ALA in the diet could block *Δ*6fad gene transcription or enzyme activity. Further, it has been shown that the amount of product generated by an enzyme is not only relative to the activity of the enzyme itself, but also the time available for the reaction [47]. We can mainly conclude on the gene expression level. Both OA and the LC n-3 PUFAs affect the genes in the pathway. An increased conversion of ALA when cells had decreased endogenous levels of DHA/increased endogenous level of OA was also seen. These findings further support the theory of the elongases as part of the regulatory steps. *elovl2* and the two *elovl5* isoforms were affected by FA treatment of the hepatocytes. In an *in vivo* study in salmon, the elongase genes also responded to changes in dietary FA composition, a significant increase of *elovl2* and *elovl5b* transcripts in the liver of vegetable oil fed fish compared to FO fed fish were seen [6]. Gregory et al. [60] found that Elovl2 converts EPA through to 24:5n-3. However, the second Elovl2 reaction (DPA to 24:5n-3) seemed to be saturated at a lower substrate concentration than the first reaction (EPA to DPA), partly explaining the increased DPA, but not DHA, often seen after certain concentrations/intakes of ALA have been exceeded. Whether the last step in the bioconversion pathway is regulated, also remains unknown. The n-3 FAs seem to increase the ACO gene expression and enzyme activity. These results agree with previous findings *in vivo*, showing both increased ACO enzyme activity and ACO gene expression when fed high DHA levels [10]. Stimulation of peroxisomal β-oxidation by increased DHA has also been confirmed in other studies with Atlantic salmon [11]. The combined activity of elovl2, ∆6fad and β-oxidation on DPA for the final production of DHA was not correlated with the substrate availability in a cell trail study by Alhazzaa et al. [47].

Taken together, the present results show the complexity in the regulation of the n-3 FA bioconversion pathway. A growing amount of evidence is confirming that the rate of PUFA synthesis will vary according to the FA composition and total PUFA content of the background diet. That *Δ6fad_a* may be regulated similarly to *Δ6fad_b*, and that *Δ6fad_c* and *Δ5fad* are regulated differently adds complexity to the picture. Levels were also reached were an increase in specific FAs given to hepatocytes not further stimulated the genes in the bioconversion pathway. The possible occurrence of rhythmic FA metabolic patterns may complicate the picture of when to get the maximal retention of health beneficial LC n-3 FAs. A deeper understanding of all the mechanisms involved in the regulation of desaturase and elongase enzyme activities is required, but when the complexity of this system is understood, better conclusions can be drawn regarding the optimal feed composition for salmon.
